# Efficacy and safety of polymer-free stent versus polymer-permanent drug-eluting stent in patients with acute coronary syndrome: a meta-analysis of randomized control trials

**DOI:** 10.1186/s12872-017-0603-5

**Published:** 2017-07-19

**Authors:** Kang Gao, Yiguang Sun, Ming Yang, Ling Han, Liwei Chen, Wenze Hu, Ping Chen, Xiaohong Li

**Affiliations:** Department of Cardiology, FuXing Hospital Affiliated to Capital Medical University, Jia #20 Street Fuxingmenwai District, Beijing, 100038 China

**Keywords:** Polymer-free stent, Polymer-based drug-eluting stent, Percutaneous coronary intervention, Meta-analysis

## Abstract

**Background:**

The efficacy and safety of polymer-free stent (PFS) versus permanent polymer drug-eluting stent (PPDES) in patients undergoing percutaneous coronary intervention (PCI) remain controversial. Our meta-analysis was undertaken to evaluate and compare the efficacy and safety of PFS with those of PPDES in patients undergoing PCI.

**Methods:**

We searched PubMed, Cochrane Library, EMBASE, and Clinical Trials.gov databases for randomized controlled trials (RCTs). The primary endpoints were incidence of stent thrombosis (ST) and target-lesion revascularization (TLR). The secondary endpoints included the incidence of major adverse cardiovascular events (MACE), myocardial infarction (MI), cardiac death (CD), late lumen loss (LLL), and diameter stenosis (DS). Subgroup analyses were also conducted based on the follow-up time.

**Results:**

Eleven RCTs met the including criteria, and 8616 patients were included in the study. No significant differences were observed between PFS and PPDES treatments in the incidence of ST (RR 0.90; 95% CI: 0.62–1.31; *P* = 0.58; *I* ^*2*^ = 0), TLR (RR 0.87; 95% CI: 0.76–1.00; *P* = 0.05; *I* ^*2*^ = 37%), CD (RR 0.89; 95% CI: 0.72–1.10; *P* = 0.28; *I* ^*2*^ = 0), MI (RR 0.87; 95% CI: 0.71–1.05; *P* = 0.15; *I* ^*2*^ = 0), LLL (SMD 0.01; 95% CI: -0.29–0.30; *P* = 0.96; I^2^ = 90%), and DS (SMD -0.01; 95% CI: - 0.25 to 0.23; *P* = 0.93; I^2^ = 83%). Meanwhile, the patients with PFS had a lower incidence of MACE (RR 0.87; 95% CI: 0.78–0.97; *P* = 0.01; *I* ^*2*^ = 0) than those with PPDES.

**Conclusion:**

In the overall analysis, patients with PFS presented a lower risk of MACE versus PPDES, but no significant difference were obtained in the risk of ST, TLR, MI, CD, DDD and DS. In the Short term follow up, patients with PSF presented a lower risk of TLR compared with PPDES.

**Electronic supplementary material:**

The online version of this article (doi:10.1186/s12872-017-0603-5) contains supplementary material, which is available to authorized users.

## Background

There are three-generation stents on the market that are used for implantation in the treatment of patients with ischemic heart disease. A representative of the first generation is the bare metal stent (BMS), whereas the drug-eluting stent (PPDES) is a second-generation stent, and the biodegradable stent is a third-generation stent [1]. Studies have shown that with the use of BMS in clinical practice, there is a certain rate of restenosis after implantation, which is usually within the range 10–30% [2]. Several concerns emerged regarding the use of PPDES, which have been associated with higher rates of late stent thrombosis (ST), mainly attributed to delayed healing and reendothelization, is due to the presence of the durable polymer coating [3]. Another type of PPDES is the polymer-free stent (PFS), which is a promising device designed to offer an attractive prospect of controlled drug-release without the potential risk of late polymer-associated adverse effects [4]. The polymer-free technology has the potential advantage to reduce the inflammatory and prothrombotic risks related to the utilization of polymers [5]. Nevertheless, no differences between PPDES and PFS in the endpoints of myocardial infarction (MI), stent thrombosis (ST), and target-lesion revascularization (TLR) were found in earlier clinical studies [6].

Our meta-analysis was undertaken to conduct a comparative evaluation of the efficacy and safety of PFS and PPDES implanted in patients with ischemic heart disease. The present meta-analysis was conducted in compliance with the Preferred Reporting Items For Systematic Review and Meta-analysis (PRISMA) [7].

## Methods

### Data sources and searches

Literature searches for RCTs were conducted in the Cochrane Library, EMBASE, PubMed, and Clinical Trials.gov databases for the period from January 1990 to September 2016. The keywords used in the literature search were “polymer-free stent”, “drug-eluting stent”, “polymer permanent”, “durable polymer”, and “percutaneous coronary intervention”. A sensitive filter for randomized controlled trials was utilized. In addition, additional references were reviewed from the bibliographies of the retrieved articles of the selected trials which were reviewed for the collection of additional information.

### Study selection

The literature records retrieved were screened by two independent investigators, and any disagreements were resolved by consultation with a senior investigator. To be included, the studies had to meet the following inclusion criteria: (1) patients undergoing PCI regardless of whether it was elective or urgent; (2) randomized controlled trials that compared PFS versus PPDES; (3) clinical outcomes were reported, such as CD ST, MI, TLR, MACE, LLL, and DS; (4) the papers were published in English. In studies with more than two arms, we used data about PFS versus PPDES. Reviews, meta-analysis, observational studies, and small-sample trials (*n* < 50) were excluded.

### Data extraction and quality assessment

Two authors extracted clinical data independently using a standardized extraction form, and other investigators were consulted to discuss and resolve any disagreement. The following information was collected from each included investigation: title or author name of the clinical investigation, publication year, baseline characteristics of participants, total number of individuals per arm, mean age, percentage of males and diabetes patients per arm, as well as data regarding the intervention and length of follow-up. The occurrence of endpoints abstracted from each study included CD, MACE, MI, TVR, and ST; mean and SD of LLL, and percent diameter stenosis. In addition, information of blinding, random sequence generation, allocation concealment, indications of incomplete outcome data, indications of selective reporting, and other forms of bias was also collected to evaluate the quality of each investigation.

### Statistical analysis

Data were analyzed according to the intention-to-treat principle. The effect size of clinical endpoints was measured by using the risk ratio (RR) with 95% confidence intervals (CIs). All *P-*values were two-tailed, with a statistical significance level limit of 0.05. Heterogeneity was assessed by the Cochran Q test and *I* ^*2*^ statistic, a Cochran’s *P* < 0.10 and *I* ^*2*^ > 50 were considered to be indicative of significant heterogeneity. Pooled analyses were conducted using the fixed-effect model, whereas the random-effect model was employed if there was heterogeneity. Publication bias was assessed using the funnel plot and Begger’s tests. The Begger’s test was not performed for the subgroup analyses due to the limited number of studies. All endpoints were reported by total and subtotal and subjected to subgroup analyses based on follow-up periods categorized in two subgroups: Short term (the follow-up is within one year) and Long term (the follow-up is longer than one year). The primary endpoint was ST and TLR, whereas the other endpoints used were secondary endpoints. Data analyses were carried out using Review Manager (RevMan) software version 5.1 (The Cochrane Collaboration, Copenhagen, Denmark). The Begger’s tests was used to evaluate the symmetry of the funnel plot were performed using STATA software version 11.1 (Stata Corp LP, College Station, TX, USA).

## Results

### Search results

**Fig. 1:**
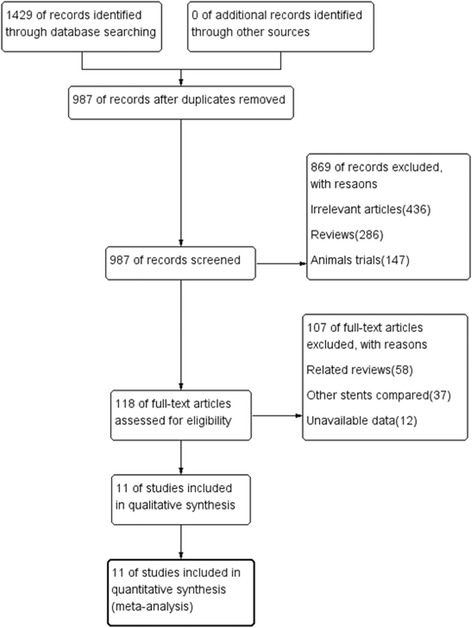
Flow chart of literature retrieval and selection

**Fig. 2:**
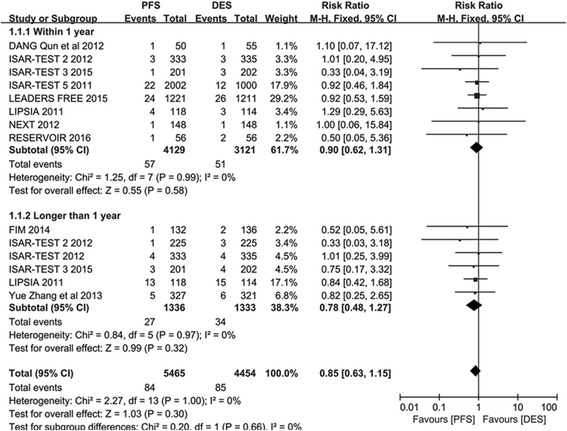
The risk ratio analysis of ST between patients with PFS and PPDES

A total of 1427 relevant publications were identified based on the search strategy, 118 of which were reviewed as full publications (Fig. 1). Eleven studies [6, 8–17] matched our selection criteria and were included in the meta-analysis. The baseline characteristics of the included studies are shown in Additional file 1: Table S1. A total of 8616 participants were included in the meta-analysis: 4813 for PFS and 3803 for PPDES. The quality assessment indicators are detailed in Additional file 1: Table S2 and Figures 1 and 2.

**Table 1:** Begger’s test result of each endpoint

Endpoint	*P* value of Begger’s test
ST	0.213
CD	0.228
MACE	0.244
MI	0.721
TLR	0.371

### Clinical Results

#### St

ST was the primary endpoint in this study. Total of 5465 participants were assigned to the PES arm group and 4454 participants to the PPDES arm group. As can be seen in Fig. 2, no significant difference in ST was detected between PFS and PPDES in the overall analysis (RR 0.85; 95% CI: 0.63–1.15; *P* = 0.30; *I* ^*2*^ = 0). Also, no significant difference was observed in the subgroup analysis between PFS and PPDES neither in the Short term subgroup (RR 0.90; 95% CI: 0.62–1.31; *P* = 0.58; *I* ^*2*^ = 0) nor in the Long term subgroup (RR 0.78; 95% CI: 0.48–1.27; *P* = 0.32; *I* ^*2*^ = 0).

#### TLR

**Fig. 3:**
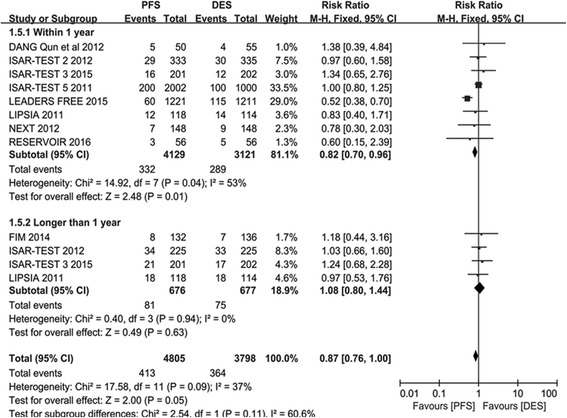
The risk ratio analysis of TLR between patients with PFS and PPDES

TLR was also a primary endpoint of our investigation. A total of 4805 participants were included in the PES arm group and 3798 participants in the PPDES arm group. A lower incidence of TLR in PFS arm than PPDES arm in the Short term analysis (RR 0.82; 95% CI: 0.70–0.96; *P* = 0.01; *I* ^*2*^ = 53%). However, as depicted in Fig. 3, when consider the overall analysis (RR 0.87; 95% CI: 0.76–1.00; *P* = 0.05; *I* ^*2*^ = 37%) and long term follow-up (RR 1.08; 95% CI: 0.80–1.14; *P* =; *I* ^*2*^ = 0), no significant was observed.

#### Mace

**Fig. 4:**
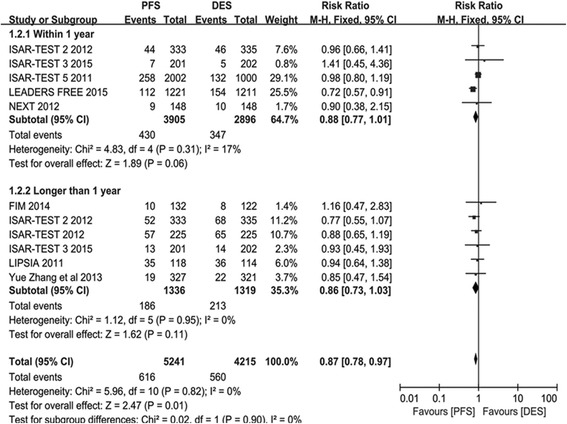
The risk ratio of MACE between patients with PFS and PPDES

There were 5241 participants assigned to PES arm and 4215 participants in PPDES arm group. The overall pooled analysis suggested that PFS decrease the risk of MACE compared with PPDES (RR 0.87; 95% CI: 0.78–0.97; *P* = 0.01; *I* ^*2*^ = 0). Whereas, there was no significant difference shown in both Short term subgroup analysis (RR 0.88; 95% CI: 0.77–1.01; *P* = 0.06; *I* ^*2*^ = 17%) and Long term subgroup analysis (RR 0.86; 95% CI: 0.73–1.03; *P* = 0.11; *I* ^*2*^ = 0). Details are shown in Fig. 4.

#### Cd

**Fig. 5:**
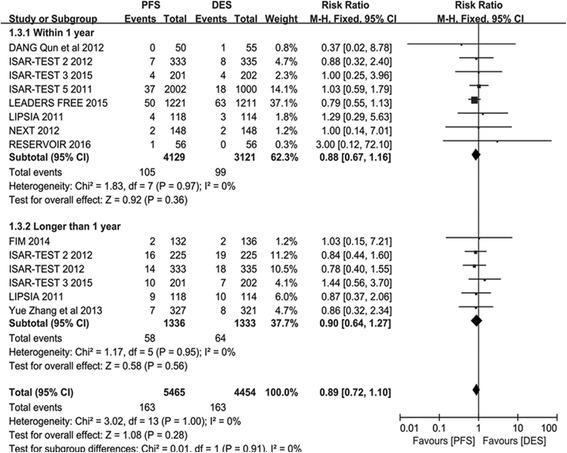
The risk ratio analysis of CD between patients with PFS and PPDES

A total of 5465 participants were included in the PES arm and 4454 participants in PPDES arm groups. The incidence of CD in PFS arm was similar to PPDES arm both in overall analysis (RR 0.89; 95% CI: 0.72–1.10; *P* = 0.28; *I* ^*2*^ = 0) and subgroup analysis (Short term subgroup: RR 0.88; 95% CI: 0.67–1.16; *P* = 0.36; *I* ^*2*^ = 0; Long term subgroup: RR 0.90; 95% CI: 0.64–1.27; *P* = 0.56; *I* ^*2*^ = 0) as shown in Fig. 5.

#### MI

**Fig. 6:**
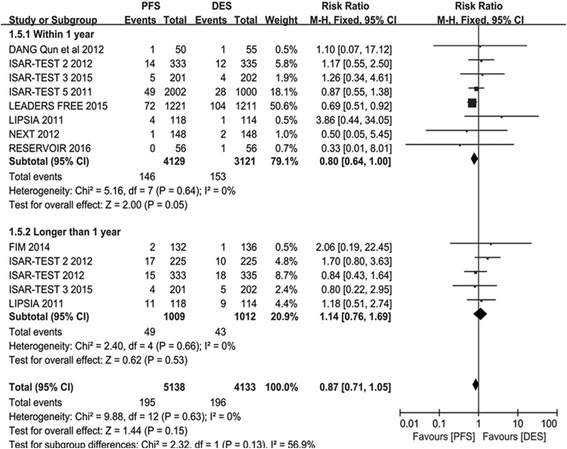
The risk ratio analysis of MI between patients with PFS and PPDES

A total number of 5138 participants were included in PES arm and 4133 participants in PPDES arm group. The incidence of MI in PFS arm was similar to PPDES arm both in overall analysis (RR 0.87; 95% CI 0.71–1.05; *P* = 0.15; *I* ^*2*^ = 0) and subgroup analysis (Short term subgroup: RR 0.80; 95% CI: 0.64–0.10; *P* = 0.05; *I* ^*2*^ = 0; Long term subgroup: RR 1.14; 95% CI: 0.76–1.69; *P* = 0.53; *I* ^*2*^ = 0). Details are displayed in Fig. 6.

#### LLL and DS

**Fig. 7:**
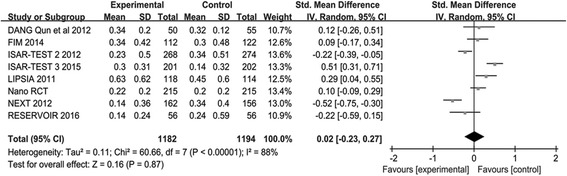
Standard mean difference (SMD) with 95% Cls of LLL

**Fig. 8:**
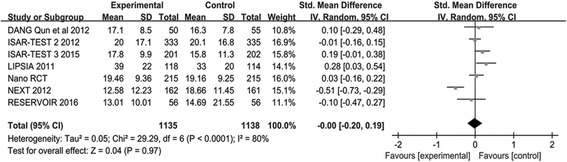
Standard mean difference (SMD) with 95% Cls of DS

The number of participants assigned to PES arm group were 967, and 979 participants in PPDES arm reported the endpoint of LLL. The meta-analysis of LLL demonstrated that PFS was associated with a similar risk compare with PPDES (SMD 0.01; 95% CI: -0.29–0.30; *P* = 0.96; I2 = 90%) as shown in Fig. 7. Total of 920 participants were assigned to the PES arm and 923 participants to the PPDES arm included for the endpoint of DS. There was no difference between PFS and PPDES in the endpoint of DS (SMD -0.01; 95% CI: - 0.25 to 0.23; *P* = 0.93; I2 = 83%) as illustrated in Fig. 8.

**Fig. 9:**
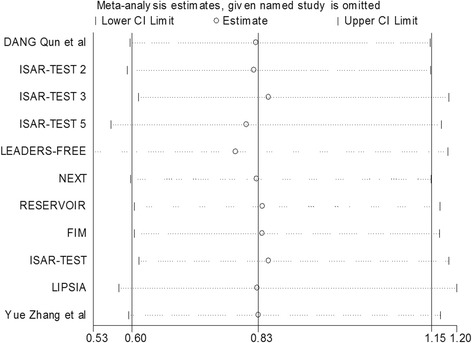
Sensitivity analysis

We obtained similar overall results after excluding each individual study (Fig. 9), which demonstrated that our study had good stability. No significant evidence of publication bias was obtained from the Begger’s test for these endpoints (Table 1).

## Discussion

Eleven investigations involving nearly 10,000 participants were included in our study to compare the efficacy and safety between PFS and PPDES. To the best of our knowledge, this is the most updated and comprehensive meta-analysis up to now. In the overall analysis of this meta-analysis, we found that the use of PFS was associated with a lower incidence of TLR and MACE than that observed in the group in which PPDES was utilized. Meanwhile, PFS decreased the risk of TLR and MI in Short term subgroup.

The first clinical investigation comparing the effects of PFS versus those of PPDES is LIPSIA [6] which reported that PFS and PPDES was associated with similar clinical results. Subsequent clinical investigations confirmed this finding, such as ISAR-TEST, [10] ISAR-TEST2, [11] and ISAR-TEST5 [13]. However, the LEADERS FREE [14] investigation indicated that PFS was associated with a lower risk of TLR, MACE, and CD compared with PPDES. With the development of scientific research, more clinical investigations compared the effects of PFS with those of PPDES emerged, and thus a meta-analysis was needed to be conducted to integrate this comprehensive information.

This is the second meta-analysis that compares the efficacy and safety of PFS with those of PPDES. The first comparative meta-analysis demonstrated that these two devices were equally effective regarding the incidence of ST, TLR, and TVR over a short and a long follow-up periods [18]. In our analysis, we found that PFS was associated with a lower incidence of TLR and MACE, and also led a continuous decrease in the incidence of TLR and MI in Short term follow-up compared with PPDES. The difference between the results of our study and those of the first meta-analysis might be due to the fact that we included data from newer clinical investigations, such as NEXT, [15] LEADERS FREE [14] and RESERVOIR [16]. Furthermore, we conducted analysis of more endpoints than this study such as the incidence of MACE, LLL and DS. In addition, clinical trials included in our study are associated with good quality and the quality of these included articles were evaluated in detail. We conducted Begger’s test and sensitivity analysis to detect the publication bias and the stability of these included investigation are very well. The findings of a pooled analysis of two RCT investigation published in 2013 which included 686 patients suggest that PFS and PPDES has similar degree of LLL at angiographic surveillance, as well as a similar risk of death, target-lesion revascularization, and MI [19]. However, this pooled analysis only reported short follow-up period and included a limited number of patients. Furthermore, publication bias and sensitivity analyses were not detected in this evaluation.

The findings of this study should be interpreted cautiously as there are some limitations that should be considered. First, due to the limited number of clinical investigations included and the small sample size, the power of our analysis is restricted. Second, the differences in patients’ clinical conditions, including drug differences, could be confusing and invalidate the results. Third, the various drug types coated with the stent in each clinical investigation might have caused heterogeneity. Finally, due to various limitations in the quality of some of the evidence, large RCTs are needed to confirm their efficacy and safety profiles of both types of stents in clinical practice.

## Conclusion

In the overall analysis, PFS is associated with a lower risk of MACE versus PPDES, but no significant difference were obtained in the risk of ST, TLR, MI, CD, DDD and DS. In the Short term follow up, PSF is associated with a lower risk of TLR compared with PPDES. These results are not absolutely beyond doubt and need to be confirmed by more high-quality RCTs.

## Additional file

Additional file 1: Table S1. Basic characteristics of the included studies. **Table S2.** Assessment of quality of included investigations. **Figure S1.** Risk of bias summary. **Figure S2.** Risk of bias graph. (DOCX 544 kb)

## Abbreviations

BMS: Bare metal stent
CD: Cardiac death
CI: Confidence interval
DES: Drug eluting stent
DS: Diameter stenosis
LLL: Late lumen loss
MACE: Major adverse cardiovascular events
MI: Myocardial infarction
PCI: Percutaneous coronary intervention
PFS: Polymer free stent
PRISMA: Preferred Reporting Items For Systematic Review and Meta-analysis
RCTs: Randomized controlled trials
RR: Risk ratio
ST: Stent thrombosis
TLR: Target lesion revascularization
