# Supplementary material for: Efficacy and safety of polymer-free stent versus polymer-permanent drug-eluting stent in patients with acute coronary syndrome: a meta-analysis of randomized control trials
Source: BMC Cardiovasc Disord. 2017 Jul 19;17:194. doi: 10.1186/s12872-017-0603-5 (PMC5518142; doi:10.1186/s12872-017-0603-5)
Supplement: Additional file 1: Table S1. — Basic characteristics of the included studies. Table S2. Assessment of quality of included investigations. Figure S1. Risk of bias summary. Figure S2. Risk of bias graph. (DOCX 544 kb) [file 12872_2017_603_MOESM1_ESM.docx]

**Supplementary Table 1. Basic characteristics of the included studies**

| Study | Year | N(PFS) | N(PPDNS) | PFS Male (%) | PPDNS Male (%) | PFS Age | PPDNS Age | PFS DM (%) | PPDNS DM (%) |
| --- | --- | --- | --- | --- | --- | --- | --- | --- | --- |
| ISAR-TEST | 2012 | 225 | 225 | 25 | 21 | 66.8±10.5 | 66.6±10.2 | 32 | 26 |
| LIPSIA | 2011 | 118 | 114 | 69 | 68 | 67.0±9.5 | 67.3±9.1 | 100 | 100 |
| ISAR-TEST 3 | 2015 | 201 | 202 | 81.7 | 98.1 | 65.0±10.7 | 66.8±9.7 | 28.7 | 26.4 |
| FIM | 2014 | 143 | 148 | 74.4 | 79.2 | 55.3±10.7 | 59.5±9.8 | 15.4 | 18.2 |
| RESERVOIR | 2014 | 56 | 56 | 19.6 | 30.4 | 66.7±9.8 | 67.2±8.8 | 100 | 100 |
| NEXT | 2012 | 162 | 161 | 76.5 | 67.7 | 64.9±10.2 | 64.4±10.5 | 29.9 | 24.2 |
| DANG Qun et al | 2012 | 50 | 50 | 70.9 | 68 | 67.1±12.5 | 65.2±13.8 | 27.3 | 24 |
| ISAR-TEST 2 | 2009 | 333 | 335 | 71.2 | 77.3 | 67.0±11.2 | 66.6±11.1 | 28.8 | 27.2 |
| Yue Zhang et al | 2013 | 327 | 321 | 65.44 | 68.54 | 65.24±10.46 | 65.87±11.1 | 25.38 | 27.73 |
| ISAR-TEST 5 | 2011 | 2002 | 1000 | 66.5 | 66.3 | 67.7±11.2 | 68.1±10.8 | 28.7 | 29.5 |
| LEADERS FREE | 2015 | 1221 | 1211 | 70.2 | 69.1 | 75.7±9.4 | 75.7±9.4 | 34 | 32.3 |

| **Supplementary Table 2. Assessment of quality of included investigations** | | | | | | | | |
| --- | --- | --- | --- | --- | --- | --- | --- | --- |
| Study | Random sequence generation | Allocation concealment | Blinding |  | Incomplete outcome data | Selective reporting | Other bias | Single/ Multicenter |
|  |  |  | participants and personnel | outcome assessment |  |  |  |  |
| ISAR-TEST | computer-generated | sealed opaque envelopes containing a computer- opaque envelopes containing a computer-generated sequence | single | blind | 0 | Low risk | Low risk | two centers in Munich, Germany |
| LIPSIA | computer-generated | Unclear | blind | blind | 2 withdrew 2 patients were randomized twice | Low risk | Low risk | Three cardiac centers in Germany |
| ISAR-TEST 3 | computer-generated | sealed opaque envelopes containing a computer- opaque envelopes containing a computer-generated sequence | single | blind | 0 | Low risk | Low risk | Two centers |
| FIM | computer-generated allocation sequence. | Unclear | single | blind | 0 | Low risk | Low risk | Multiple |
| RESERVOIR | computer-generated sequence | Unclear | single | blind | 0 | Low risk | Low risk |  |
| NEXT | computer-generated blocked randomization list | Allocation was insured by sequentially numbered and sealed envelopes | single | blind | In 6 months, 4 withdraw; 1 died 4 withdraw | Low risk | Low risk | 11 centers |
| DANG Qun et al | computer-generated | sealed opaque envelopes containing a computer- opaque envelopes containing a computer-generated sequence | unclear | blind | 0 | Low risk | Low risk | single |
| ISAR-TEST 2 | computer-generated | sealed opaque envelopes containing a computer- opaque envelopes containing a computer-generated sequence | blind | blind | 125 no-follow for angiogram | Low risk | Low risk | three limus agent stents |
| Yue Zhang et al | computer-generated | sealed opaque envelopes containing a computer- opaque envelopes containing a computer-generated sequence | unclear | blind | 17 patients were lost to follow-up  2 patients withdrew | Low risk | Low risk | five participating centres |
| ISAR-TEST 5 | computer-generated | sealed opaque envelopes containing a computer- opaque envelopes containing a computer-generated sequence | single | blind | 43 withdraw 14 withdraw | Low risk | Low risk | 2 participating centers |
| LEADERS FREE | Web-based or telephone interactive voice response system | packaged and identified by serial number | double | blind | 23 withdrew before 12-mo visit 24 were lost to follow-up | Low risk | Low risk | 4 continents |


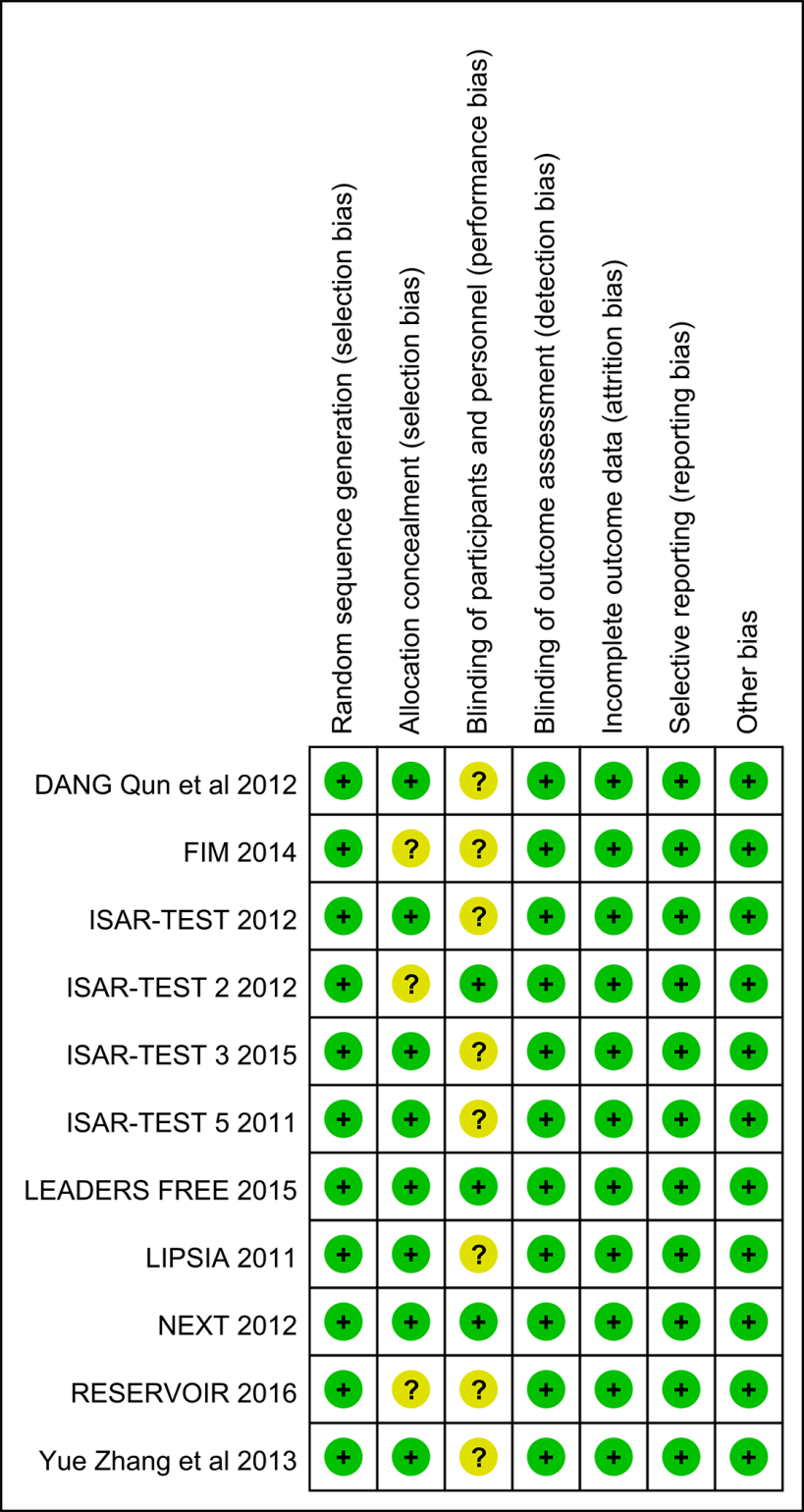


**Supplementary Figure 1. Risk of bias summary**


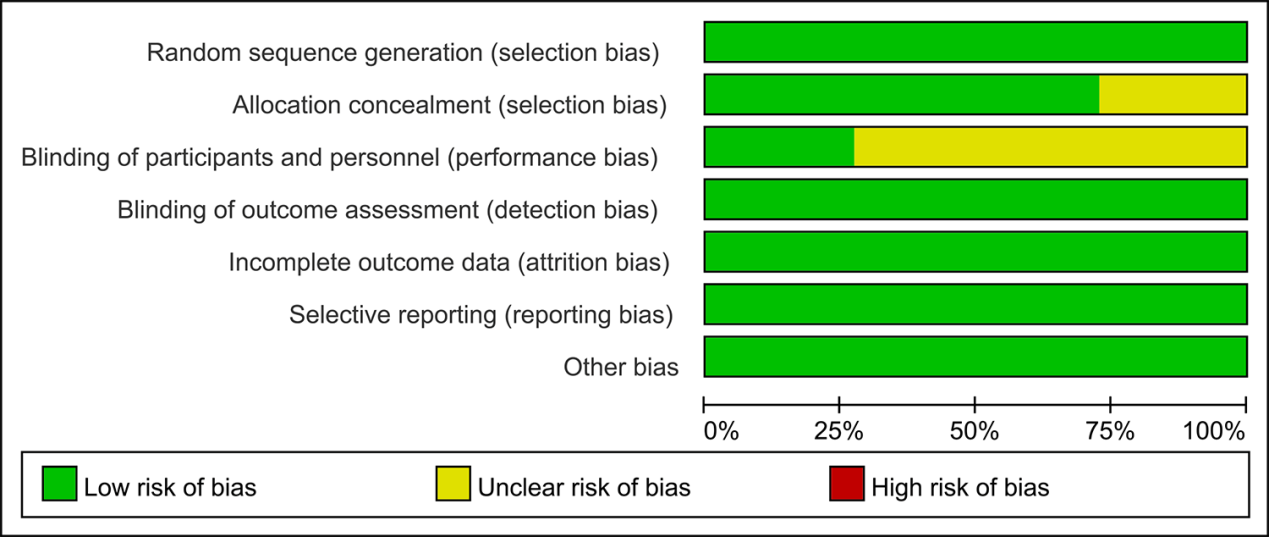


Supplementary Figure 1. Risk of bias graph
